# Endoscopic fenestration of an enlarging giant occipital arachnoid cyst

**DOI:** 10.3171/2023.1.FOCVID22129

**Published:** 2023-04-01

**Authors:** Rachel Blue, Jaskeerat Gujral, Hasan S. Ahmad, Omar Choudhri

**Affiliations:** 1 Department of Neurosurgery, University of Pennsylvania, Philadelphia; and; 2 University of Pennsylvania, College of Arts and Sciences, Philadelphia, Pennsylvania

**Keywords:** endoscopic, fenestration, arachnoid cyst, ventricle

## Abstract

This case demonstrates an endoscopic fenestration of an enlarging giant occipital arachnoid cyst. The patient is a 42-year-old woman presenting with headache, progressive vision loss, and nausea and vomiting. MRI demonstrates a large, nonenhancing cystic lesion in the right occipital lobe measuring up to 8.3 cm, consistent with an arachnoid cyst. This surgical video illustrates the technique for an endoscopic fenestration into the native ventricular system utilizing stereotactic MRI-guided stealth navigation. Postoperatively, the patient had full recovery with improvement of headaches and vision and was discharged on postoperative day 1 without complications.

The video can be found here: https://stream.cadmore.media/r10.3171/2023.1.FOCVID22129

## Transcript

### 0:20 Case History.

This is a surgical video demonstrating endoscopic fenestration of an enlarging right occipital arachnoid cyst in a lady in her 40s with worsening headaches, as well as visual field loss and optic disc swelling.

### 0:35 Imaging and Surgical Options.

The cyst measured approximately 8 cm with progressive enlargement over the years between 2010 and 2021, which had started significant mass effect and displacement of the ventricular system as well as of the occipital and temporal lobes. Given her worsening symptoms, the cyst, which is now being seen in different views, including the axial, coronal, and sagittal views, was considered for surgical intervention. The options considered were placement of a cystoperitoneal shunt, craniotomy for release of the cyst contents, as well as endoscopic fenestration.^1–5^ The shunt was not the first choice, given the need to leave permanent hardware.

### 1:21 Surgical Plan.

The surgical plan included having the patient prone, the use of stereotactic navigation, and using an endoscope on a preplanned trajectory, as shown below, to navigate down to the ventricle and to fenestrate the cyst.

### 1:35 Stereotactic Trajectory and Opening of Cyst Wall.

Here the endoscope is being introduced to the burr hole and we’re using electrocautery to find the spot, based on navigation, where the cyst contents come closest to the ventricular system. After using electrocautery to create an entry point, alligator forceps are used to enlarge the opening of the cyst to create the maximal opening and connection between the cyst as well as the ventricular system. This is a technique that is used sequentially to provide the biggest window of access, and after the first entry point, we use electrocautery, joining the area to further increase the size of this fenestration site, which is being seen over here. After electrocautery, we will follow that up with an alligator forceps clamp to further enlarge the created opening, and irrigation is constantly used, which provides a visual and tactile feel of the success of the fenestration depending on the billowing and movement of the cyst wall contents, which often indicates flow of fluid and creation of a connection.

### 2:50 Expansion of Cyst Wall Opening.

Microscissors are here being used to take down additional tissue between the two areas of electrocautery and the site of fenestration to maximize the creation of a bigger opening and chances of success. And again, this is the irrigation that is demonstrating the movement of the cyst walls and the flow of fluid from the cyst into the ventricular system. It is hard to understand the anatomy when inside a giant occipital arachnoid cyst, given the lack of anatomical landmarks and using navigation is helpful in understanding these locations. These thin cyst walls are further being divided with the use of microscissors to continue enlarging the site of opening at the fenestration. This further highlights the need for high-resolution endoscopy and accurate targeting in completion of some of this endoscopic microsurgical work that allows us to create these passages of fenestration for these lesions.

### 4:00 Hemostasis.

Hemostasis is achieved with constant irrigation to stop any bleeding and to promote formation of blood clots, and a ventricular drain is placed under vision through the area of fenestration, which helps in the clearance of blood products, for keeping the fenestration open in the first 24 hours and also for monitoring of postoperative intracranial pressures at the site of fenestration.

### 4:27 EVD Placement.

Placement of this ventricular drain under vision allows for safe, accurate placement of these monitoring devices.

### 4:37 Postoperative Imaging and Recovery.

This is the imagining preoperatively and postoperatively, approximately 3 months apart, that demonstrates decrease in the size of the cysts and reconstitution of normal ventricular anatomy. The patient was discharged the next postoperative day, had improvement of headaches, and the visual improvement continued at 2 years’ follow-up, with improvement in the optic disc edema as well as the visual fields seen by the ophthalmologist.

### 5:05 Key Learning Points.

The occipital arachnoid cysts are rare lesions, but they can be symptomatic, especially with massive enlargement, as in this case. The treatment can be targeted and precise, using stereotactic navigation and high-resolution endoscopy, which allows us to make a large enough opening for accurate rerouting of cyst fluid into the ventricular system, and use of an external ventricular drain is useful for the reasons mentioned before. These are the references, thank you.

## Disclosures

The authors report no conflict of interest concerning the materials or methods used in this study or the findings specified in this publication.

## Author Contributions

Primary surgeon: Choudhri. Assistant surgeon: Blue. Editing and drafting the video and abstract: all authors. Critically revising the work: Ahmad, Choudhri. Reviewed submitted version of the work: Blue, Choudhri. Approved the final version of the work on behalf of all authors: Blue. Supervision: Choudhri.

## Supplemental Information

### Patient Informed Consent

The necessary patient informed consent was obtained in this study.
